# *In Vitro* Evaluation of Antioxidant and Antimicrobial Activities of* Melaleuca alternifolia* Essential Oil

**DOI:** 10.1155/2018/2396109

**Published:** 2018-05-06

**Authors:** Xiaofeng Zhang, Yanjun Guo, Liying Guo, Hui Jiang, Qianhua Ji

**Affiliations:** ^1^Fruit Tree Research Institute, Zhaoqing University, Zhaoqing 526000, China; ^2^College of Life Science, Zhaoqing University, Zhaoqing 526000, China

## Abstract

The* in vitro* antioxidant and antimicrobial activity of the essential oil from* Melaleuca alternifolia (M. alternifolia)* was evaluated in this report. The antioxidant potential of the essential oil from* M. alternifolia* was evaluated by the DPPH (2,2-diphenyl-1-picrylhydrazyl) method, thiobarbituric acid reactive species (TBARS) assay, and the hydroxyl radical scavenging activity method. The essential oil from* M. alternifolia *was able to reduce DPPH with an EC50 (concentration for 50% of maximal effect) of 48.35 *μ*g/ml, inhibit the lipid peroxidation with an IC50 (50% inhibitory concentration) of 135.9 *μ*g/ml, and eliminate hydroxyl radicals with an EC50 of 43.71 *μ*g/ml. Antimicrobial screening, minimum inhibitory concentration, and minimum bactericidal concentration assays showed that the essential oil from* M. alternifolia* inhibited strongly the growth of different types of microorganisms, including* Escherichia coli*,* Staphylococcus aureus*,* Pseudomonas aeruginosa*,* Penicillium italicum Wehmer,* and* Penicillium digitatum Sacc*. Thus, the essential oil of* M. alternifolia* possesses antioxidant and antimicrobial activity and could be suitable for use as a natural preservative ingredient in food, agriculture, and pharmaceutical industries.

## 1. Introduction

In the last decade, research into essential (volatile) oils has received increasing attention from both industrial and academic sectors because of the growing interest in green consumerism and the need for alternative techniques to assure the quality and safety of perishable foods [1]. The plant* Melaleuca alternifolia (M. alternifolia)* belongs to the Myrtaceae family [2], which is described as a scrubland species and found throughout South America, western India, and Australia [3]. The plant has been used in medicine, cosmetics, food, agriculture, and other industries [4–7]. In recent years,* M. alternifolia* has also been gradually introduced in southern China. The essential oil of* M. alternifolia* is derived by steam or hydrodistillation from* M. alternifolia* as secondary metabolites and is monoterpene-rich, volatile oil characterized by a strong odor [8, 9].

Several studies conducted on natural plant essential oils have indicated that these oils may be used as antimicrobial agents and have potential use in industrial applications [10–12].* M. alternifolia*, also known as tea tree, has been investigated as an alternative antimicrobial agent [13]; however, there is no information that focuses exclusively on the antioxidant activities of the essential oil from* M. alternifolia*. The purpose of the present study was to evaluate the* in vitro* antioxidant and antimicrobial activity of the essential oil from* M. alternifolia*.

## 2. Materials and Methods

### 2.1. Plant Material

*Melaleuca alternifolia (M. alternifolia)* samples were collected in four locations of Zhaoqing City of Guangdong Province at altitudes between 300 and 600 m. Samples were dried in well-ventilated spaces away from sunlight. Samples were then placed at 50°C to dry the samples to a constant, dry weight and disintegrated through a 90-mesh sieve. Air-dried plant materials were hydrodistilled using a Clevenger-type apparatus. The composition of* M. alternifolia* essential oil was analyzed in the laboratory using Hewlett-Packard 6890 gas chromatograph equipped with a cross-linked 5% PH ME siloxane Hewlett-Packard-5MS capillary column (25 m × 0.25 mm ID, 0.25 *μ*m film thickness), coupled to a Hewlett-Packard 5972A mass spectrometer (Hewlett-Packard Ltd., Bracknell, UK). The GC operating conditions were as follows: helium as carrier gas with a flow rate 2.0 ml/min; column temperature programming from 60°C to 275°C at 4°C/min; injector and FID detector temperatures of 215 and 275°C, respectively. The MS operating parameters were as follows: ionization potential, 70 ev; resolution, 1000; ion source temperature, 250°C. Identification of components was based on GC retention indices and the fragmentation patterns of the mass spectra with those of authentic samples, as well as the NIST 98 and HPCH 2205 GC–MS libraries. Relative percentage amounts were obtained directly from GC peak areas. The composition of the essential oil from* M. alternifolia* is presented in Table 1.

### 2.2. Antioxidative Activity

#### 2.2.1. DPPH Assay

This spectrophotometric assay uses the stable 2,2-diphenylpicrylhydrazyl (DPPH) radical as a reagent [14]. One milliliter of various concentrations of the samples in methanol was added to 3 mL of 100 *μ*M DPPH dissolved in methanol. After incubation for 30 min at room temperature, the absorbance was measured against a blank at 517 nm. The antioxidant activity was determined using the following equation: (%, elimination of DPPH) = (*A*_blank_ − *A*_sample_/*A*_blank_) × 100. The extract concentration providing 50% elimination (EC50) was calculated from the graph plotting inhibition percentage against extract concentration. Tests were carried out in triplicate.

#### 2.2.2. TBARS Assay

The thiobarbituric acid reactive substances (TBARS) method was used as described by [15]. One milliliter of various concentrations of the samples in methanol was added to 3 mL of 10% yolk homogenate and incubated for 30 min at room temperature. Then, 0.1 mL of a ferrous sulfate solution was added and the reaction incubated for 30 min at 37°C. Next, 3.0 mL of 10% trichloroacetic acid was added and the reaction centrifuged at 2000*g* for 5 min. Subsequently, 1.0 mL of the supernatant was mixed with 3.0 mL of a 0.67% TBA solution in 50% glacial acetic acid. The mixture was heated in a boiling water bath for ~30 min. The reaction mixture was centrifuged at 2000*g* for 5 min if the solution became turbid. Finally, the absorbance was measured at 535 nm. A decrease in absorbance indicates an increase in antioxidant activity. The antioxidant activity was determined using the following equation: (%, inhibition of peroxidation) = (*A*_blank_ − *A*_sample_/*A*_blank_) × 100. The extract concentration providing 50% inhibition (IC50) was calculated from the graph plotting inhibition percentage against extract concentration. Tests were carried out in triplicate.

#### 2.2.3. Hydroxyl Radical Scavenging Activity

The hydroxyl radical scavenging activity was measured by the method described in [16]. The reaction mixture (1.0 mL) consisting of 0.1 mL of 2-deoxy-D-ribose (28 mM in 20 mM KH_2_PO_4_-KOH buffer, pH 7.4), 0.1 mL of various concentrations of the samples, 0.2 mL EDTA (1.04 mM) and 200 *μ*M FeCl_3_ (1 : 1 v/v), 0.1 mL of H_2_O_2_ (1.0 mM), and 0.1 mL ascorbic acid (1.0 mM) was incubated at 37°C for 1 h. One milliliter of thiobarbituric acid (1%) and 1.0 mL of trichloroacetic acid (2.8%) were added and the solution incubated at 100°C for 20 min. After cooling, the absorbance was measured at 532 nm. The antioxidant activity was determined using the following equation: (%, elimination of hydroxyl radical) = (*A*_blank_ − *A*_sample_/*A*_blank_) × 100. The extract concentration providing 50% elimination (EC50) was calculated from the graph plotting inhibition percentage against extract concentration. Tests were carried out in triplicate.

### 2.3. Antibacterial Activity

#### 2.3.1. Microbial Strains

*Escherichia coli* ATCC25922* (E. coli), Staphylococcus aureus* ATCC25923* (S. aureus)*,* Pseudomonas aeruginosa* ATCC27853* (P. aeruginosa)*,* Penicillium italicum Wehmer (P. italicum Wehmer),* and* Penicillium digitatum Sacc. (P. digitatum Sacc.)* were obtained from the China Center for Type Culture Collection (Wuhan University, China).

#### 2.3.2. Antimicrobial Screening

Paper disks with 6 mm diameter were soaked with 0.1 mL of the essential oil from* M. alternifolia* and placed on the surface of solid media plates previously inoculated with the different microorganisms (6.0 log⁡CFU/mL) tested in this study.* P. italicum Wehmer* and* P. digitatum Sacc. *were cultured at 28°C and 120 rpm for 48 h, whereas* E. coli*,* S. aureus, *and* P. aeruginosa *were cultured at 37°C and 120 rpm for 24 h. The size of the halo for each microorganism was recorded by measuring the zones of growth inhibition surrounding the disks. Individual samples were examined in triplicate. The size of the halos is presented as means ± standard deviation.

#### 2.3.3. Minimum Inhibitory Concentration (MIC) and Minimum Bactericidal Concentration (MBC)

A broth microdilution method was used to determine the MIC and MBC. The potato dextrose broth and Mueller Hinton broth were prepared at twice the final concentration.* M. alternifolia *essential oil was added to glass tubes to yield final sample media concentrations at 0.2 to 48 mg/mL for the potato dextrose and Mueller Hinton broths, respectively. The control sets were run simultaneously without the addition of an antimicrobial agent. An appropriate volume of inoculum (6.0 log⁡CFU/mL) was added to the media to give an approximate final cell concentration of 4.0 log⁡CFU/mL.* P. italicum Wehmer* and* P. digitatum Sacc. *cultures in potato dextrose broth were incubated at 28°C and 120 rpm for 48 h, whereas* E. coli* and* S. aureus *cultures in Mueller Hinton broth were incubated at 37°C and 120 rpm for 24 h. The essential oil concentration that yielded no visible growth for a tested species was considered the MIC for that particular species. Then, 0.1 mL suspension obtained from the above transparent tubes was spread onto either potato dextrose agar (PDA) or Mueller Hinton agar (MHA). After 48 h culturing at 28°C (fungi) or 48 h culturing at 37°C (bacteria), the concentration of inoculated suspension containing the lowest level of antimicrobial agent that showed no colonies on the plate was determined as the MBC.

## 3. Results and Discussion

### 3.1. Chemical Composition of the Melaleuca alternifolia Essential Oil

The essential oil from* M. alternifolia *can be obtained easily from the hydrodistillation of* M. alternifolia *leaves, and the chemical composition is dependent on the extraction method used and crop region the samples are taken from [3]. The chemical components of* M. alternifolia *essential oil used in this study are presented in Table 1. We identified 14 components representing 92.87% of the oil. Terpinene-4-ol (31.11%), *γ*-terpinene (25.30%), and *α*-terpinene (12.70%) were the major constituents followed by 1,8-cineole (6.83%), *ρ*-cymene (4.23%), terpinolene (4.03%), limonene (2.50%), *α*-terpineol (2.35%), aromadendrene (1.75%), and *δ*-cadinene (1.41%). As alluded to above, the composition of* M. alternifolia *essential oil is influenced by several factors, including local, climatic, seasonal, and experimental conditions. Thus, the biological activities of each essential oil preparation will likely vary [17].

### 3.2. Antioxidant Activity

**Figure 1:**
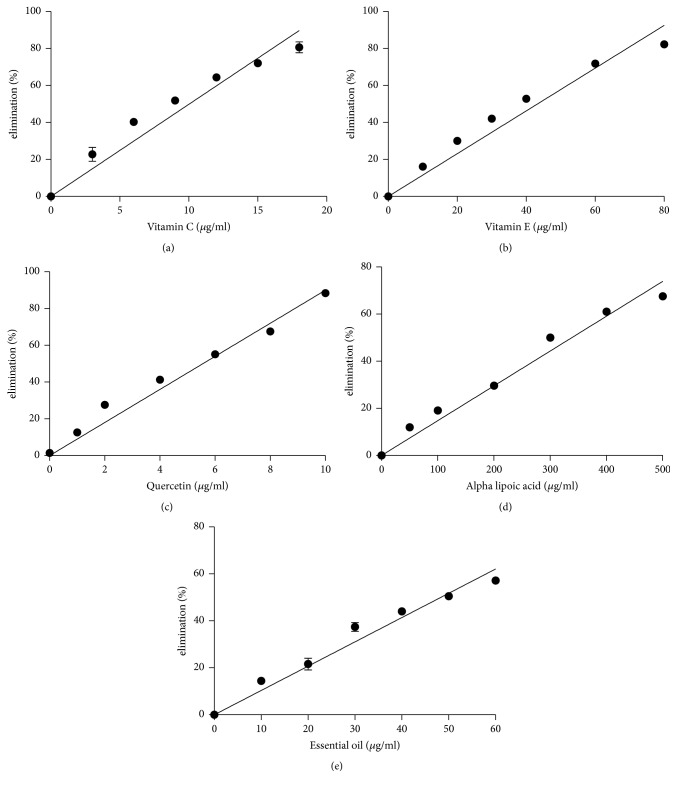
Antioxidant activity of different antioxidants by DPPH assay. (a) Vitamin C; (b) vitamin E; (c) quercetin; (d) alpha lipoic acid; and (e)* Melaleuca alternifolia* essential oil. Values represent means ± SEM, *n* = 3.

**Figure 2:**
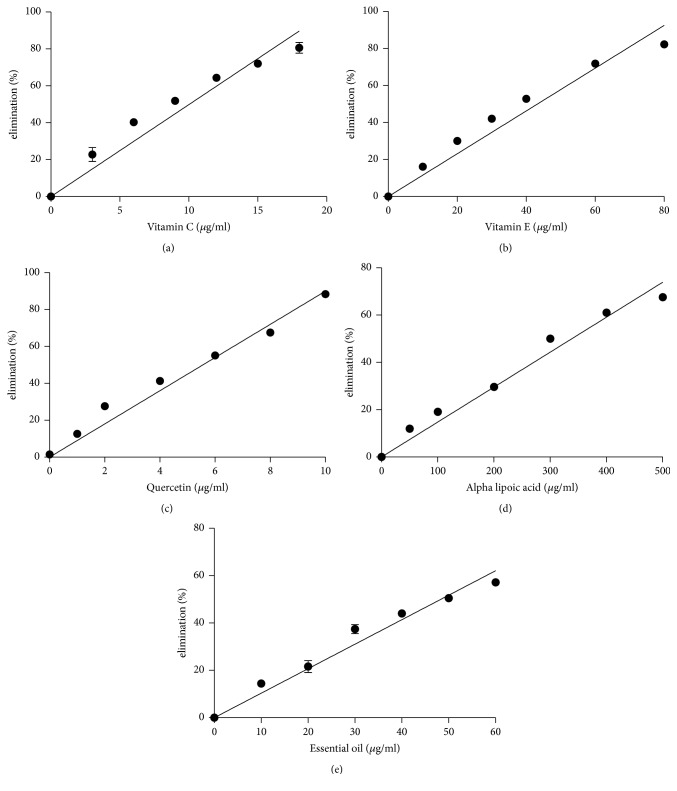
Antioxidant activity of different antioxidants by hydroxyl radical scavenging activity. (a) Vitamin C; (b) vitamin E; (c) quercetin; (d) alpha lipoic acid; and (e)* Melaleuca alternifolia* essential oil. Values represent means ± SEM, *n* = 3.

**Figure 3:**
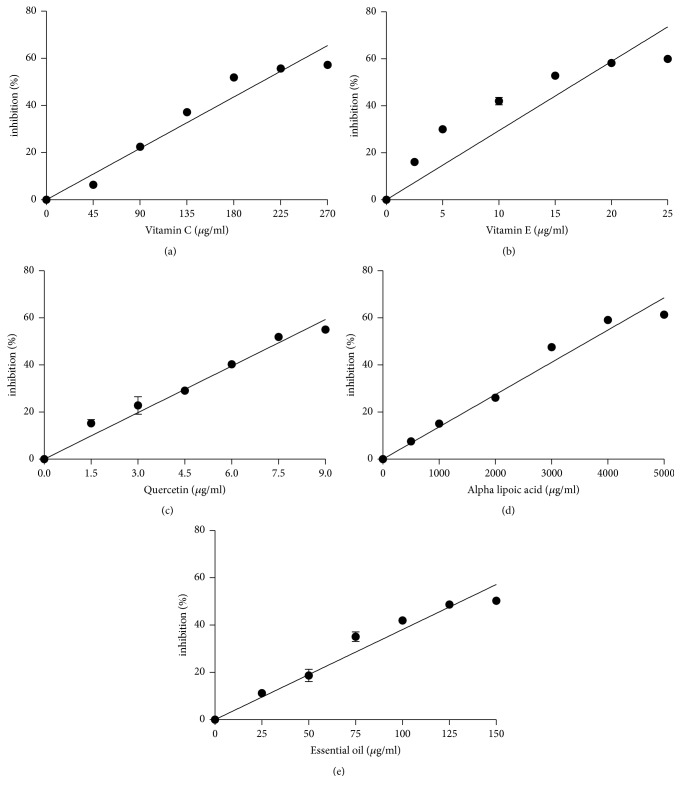
Antioxidant activity of different antioxidants by TBARS method. (a) Vitamin C; (b) vitamin E; (c) quercetin; (d) alpha lipoic acid; and (e)* Melaleuca alternifolia* essential oil. Values represent means ± SEM, *n* = 3.

As previously described, the use of different methods is required when assessing antioxidant activity [18]. In this study, the DPPH assay, hydroxyl radical scavenging properties, and inhibition effects on lipid peroxidation were used to examine the* M. alternifolia *essential oil antioxidant activity. Vitamin C, vitamin E, quercetin, and *α*-lipoic acid are four known natural antioxidants and were used as positive controls. The DPPH assay has been used widely in determining the free radical scavenging properties of plant extracts [15].  Figure 1 and Table 2 show the results of the DPPH assay. The results showed that the antioxidant power decreased in the order quercetin ≫ vitamin C > vitamin E >* M. alternifolia *essential oil > *α*-lipoic acid. Reactive oxygen species, in particular hydroxyl radicals, are implicated in oxidative damage to fatty acids, DNA, proteins, and other cellular components [19]. Hydroxyl radicals are the most potent oxidizing species of biological membrane proteins and lipids and have an extremely short half-life [20]. The results of the hydroxyl radical scavenging activity test for the* M. alternifolia *essential oil are similar to the DPPH assay results. Here, the antioxidant power decreased in the order quercetin > vitamin C >* M. alternifolia *essential oil > vitamin E > *α*-lipoic acid (Figure 2 and Table 2). Nonetheless, the hydroxyl radical scavenging activity test showed that the antioxidant activity of* M. alternifolia *essential oil is stronger than *α*-tocopherol. The TBARS assay is used widely to measure lipid oxidation and antioxidant activity [21] and was used here to measure the amount of lipid degradation [15] in the presence of the essential oil from* M. alternifolia*. The inhibition of lipid peroxidation by the* M. alternifolia *essential oil was examined and the results compared with those for quercetin, vitamin C, vitamin E, and *α*-lipoic acid. As shown in Figure 3 and Table 2, the antioxidant power decreased in the order quercetin > vitamin E >* M. alternifolia *essential oil > vitamin C > *α*-lipoic acid. In the present study, we found that, compared with known natural antioxidants, the essential oil from* M. alternifolia* exhibited strong free radical scavenging properties and inhibited lipid peroxidation. These attributes are probably because of the inherent activity of some of the components present in the essential oil, in particular the phenols, which inhibit or reduce the rate of aerobic oxidation of organic matter [22]. In fact, it has widely been demonstrated that monoterpene hydrocarbons are better antioxidant compounds with respect to sesquiterpenes and particularly those with strongly activated methylene groups in their structure, such as terpinene-4-ol, *γ*-terpinene, and *α*-terpinene, were the most active [23].

### 3.3. Antibacterial Activity

*E. coli, S. aureus, P. aeruginosa, P. italicum Wehmer, and P. digitatum Sacc.* are different types of microorganisms.* E. coli, S. aureus,* and* P. aeruginosa* are pathogenic bacteria [24], whereas* P. italicum Wehmer* and* P. digitatum Sacc.* are fungi [25]. Because these organisms infect, damage, and destroy material used in manufacturing goods or infect the goods produced; these microorganisms increase economic costs in various industries, including medicinal, cosmetic, agriculture, and food industries. Thus, the antibacterial activity of the essential oil from* M. alternifolia* was investigated with three different methods: the antimicrobial screening method and the MIC and MBC assays. The results presented in Table 3 clearly show that the essential oil of* M. alternifolia *displayed significant antimicrobial activity against all microorganisms tested. The Gram-positive bacteria were more sensitive to the essential oil than Gram-negative bacteria and fungi.* E. coli* had the lowest MIC (2 mg/ml). These data are consistent with previous observations that* Melaleuca alternifolia *essential oil possesses antibacterial and antifungal activity [11]. According to Cox et al. (2000), the antimicrobial activity of the* M. alternifolia* terpenes is associated with their strong hydrophobicity. Here, the hydrophobic terpenes interact strongly with the membrane lipids of the pathogenic microorganisms, which affect the permeability of the membrane [10]. Among the main consequences of membrane permeability are (i) modification of the proton-motive force, leading to a deficit in the production of cellular energy caused by the decrease in ATP generation, and (ii) cellular lyses due to leakage or coagulation of the cytoplasm [26, 27]. The compound terpinene-4-ol, also identified amongst the main constituents of the Iranian Cymbopogon Olivieri essential oil, has been implicated in the antimicrobial activity against Gram-positive bacteria, Gram-negative bacteria, and the yeast* Candida albicans* [28].

## 4. Conclusions

In conclusion, our study is the first report describing the* in vitro* antioxidant properties of the essential oil from* M. alternifolia*. Because of its strong antibacterial and excellent protective features exhibited in antioxidant activity tests, this essential oil and extracts from the herbal parts of* M. alternifolia* represent a potential natural source that can be used freely in food, agriculture, and pharmaceutical industries as a culinary herb.

## Figures and Tables

**Table 1:** Chemical composition of *Melaleuca alternifolia* essential oil.

Components^a^	Composition%
Terpinene-4-ol	31.11
*γ*-Terpinene	25.30
*α*-Terpinene	12.70
1,8-Cineole	6.83
*ρ*-Cymene	4.23
Terpinolene	4.03
Limonene	2.50
*α*-Terpineol	2.35
Aromadendrene	1.75
*δ*-Cadinene	1.41
Sabinene	0.28
Globulol	0.24
Viridiflorol	0.14
Total	92.87

^a^The dates were provided by Meriden Animal Health Lt.

**Table 2:** Antioxidative activity of the essential oil of *Melaleuca alternifolia*.

Sample (*μ*g/mL)	Test system		
	DPPH assay (EC50)	Hydroxyl radical scavenging activity (EC50)	TBARS method (IC50)
Vitamin C	7.79	7.43	185.7
Vitamin E	34.59	113.1	14.19
Quercetin	4.46	9.64	7.82
Alpha lipoic acid	305.6	1877	3414
*Melaleuca alternifolia* essential oil	48.35	43.71	135.9

**Table 3:** Antimicrobial activity of the essential oil of *Melaleuca alternifolia*.

Microorganisms	*Melaleuca alternifolia* essential oil		
	DD (mm)	MIC (mg/mL)	MBC (mg/mL)
*Escherichia coli*	12 ± 1.63	8	8
*Staphylococcus aureus*	26 ± 2.80	2	2
*Pseudomonas aeruginosa*	10 ± 0.94	12	12
*Penicillium italicum Wehmer*	9 ± 0.41	12	12
*Penicillium digitatum Sacc.*	8 ± 0.47	24	24

DD, diameter of zone of inhibition (mm) including disc diameter of 6 mm.
